# Innovative approaches in discussions of diabetes among healthcare sector actors in Germany

**DOI:** 10.1057/s41271-024-00509-x

**Published:** 2024-07-13

**Authors:** Sabahat Ölcer, Maike Scheipers, Manfred Erbsland, Constanze Sharma

**Affiliations:** 1 Clinic for Palliative Medicine, University Medical Center Göttingen, University of Göttingen, Göttingen, Germany; 2 Department of Human-Centred Technological Development, Institute of Computer Science, Ruhr West University of Applied Sciences, Campus Bottrop, Germany; 3 Institute for Management, Economics and Care in the Health Sector, Ludwigshafen University of Business and Society, Ludwigshafen am Rhein, Germany

**Keywords:** Diabetes, Healthcare sector, World Café method, Awareness, digital solutions, Prevention

## Abstract

The dramatic growth in the rate of diabetes prompts serious debates about awareness, early diagnosis, and care interventions. This Viewpoint aims to explore, from the perspectives of healthcare sector representatives, what challenges and difficulties they face in dealing with diabetes and how these can be overcome. We applied the World Café method for group discussions, which enabled us to bring together 50 stakeholders. They identified challenges at institutional and structural levels under the concepts of awareness, digitalisation, and new forms of care and shared their suggestions for feasible solutions. We learned there is a need for a diabetes map of Germany to identify risk groups and that hybrid solutions should be implemented for treatment, care, prevention, and early diagnosis, considering digital infrastructure. Also, the demand for digital innovation in the healthcare system raised concerns about information transparency and data management.

**Supplementary Information:**

The online version contains supplementary material available at 10.1057/s41271-024-00509-x.

## Key messages

- Use of transparent information and data management policies builds a stronger sense of trust between beneficiaries and service providers.
- Digital innovations in healthcare systems require hybrid solutions in the short-term.
- Social media campaigns can be effective for raising awareness and better understanding of diabetes.

**Supplementary Information:**

The online version contains supplementary material available at 10.1057/s41271-024-00509-x.

## Introduction

Diabetes mellitus is one of four major non-communicable diseases, resulting in 6.7 million deaths worldwide in 2021 [1]. This global epidemic is a cause for concern, due to increased health expenditures, emerging associated diseases, its impact on quality of life, and the restrictions it imposes on everyday life. According to clinical data, diabetes is one of the most common chronic diseases in Germany and demonstrates an increasing incidence and prevalence [2]. The National diabetes surveillance report of 2019 estimated the number of people with diabetes in Germany to be 7.5 million [3], with total diabetes-related direct health expenditure around €7.4 billion in 2020 [4]. Most diabetic patients—about 93%—are affected by Type 2 diabetes [5]. Experts envisage that the number of people with Type 2 diabetes in Germany could rise to 12 million by 2040 [2, 5]. Ageing of the population, delayed diagnosis of prediabetes, and changing lifestyles may explain this rapid increase [6]. To prevent the disease, the World Health Organization recommends lifestyle changes, such as healthy eating, exercising regularly, and avoiding weight gain and smoking [7]. These recommendations, however, may not always be sufficient to avoid this complex chronic disease.

The public and private healthcare sectors engage in various efforts to prevent diabetes, reduce its effects, alleviate the workload of healthcare professionals, minimise health expenditures with effective strategies, and to improve the quality of life of patients with diabetes. Awareness [8], digitalisation [9], and the creation of new forms of care [10] are dominant activities in diabetes care. However, important institutional and structural challenges remain. Previous research may not provide sufficient insight into the efforts of the healthcare industry in Germany, particularly from the perspective of actors in the sector dealing with diabetes.

Thus, we explored what challenges and difficulties arise at the institutional and structural levels in tackling diabetes and how they can be overcome. With the participation of relevant actors from the fields of business, care, and science in focus group discussions (the World Café method), we aimed to obtain a holistic picture of the discussions about diabetes and insights from them.

## Methods

### Study design

We drew data from a formal research project focused on diabetes in the German state of Rhineland-Palatinate. We applied the World Café method [11] of focus group discussions to explore what challenges and difficulties complicate efforts to diminish the impact of diabetes at the institutional and structural levels, and how these can be overcome. We chose the World Café method (with participants at metaphorical tables of a café) to enable us to bring small groups of people together to discuss diabetes, prompt new connections among players in the sector, and insights from more collective thinking. We formulated our questions for these discussions around three concepts: awareness, digitalisation, and new forms of care.

### Participant recruitment

We selected potential participants among a wider pool of stakeholders through the network of the cooperation partner of the project. The cooperation partner was a non-profit platform that contributed to the growth of the healthcare sector in the region. Its members included companies, suppliers, and institutions. We were able to identify appropriate stakeholders through purposive sampling by considering the variety of entities and the roles of those working in this sector, such as health insurance companies, the pharmaceutical industry, drug stores, publishers, healthcare professionals, nutrition, and scientists. The stakeholders’ own networks expanded our sample. We selected attendees who were well-informed about diabetes and capable of taking on relevant responsibilities in their current positions. We sought to assemble a group (as to their knowledge, capabilities, and responsibilities) representing a diverse array of institutional players in the industry. We contacted potential attendees via email to request their participation and provided them with information about the objectives, discussions, and a consent form. Attendees also signed a form during registration giving permission for the sessions to be digitally recorded and analysed. Attendance was voluntary, and stakeholders did not receive any remuneration or reward.

We invited 76 stakeholders, 50 of whom participated in the discussions (Table 1). Companies included developers, producers and manufacturers of medical devices, as well as manufacturers of diagnostic tests such as glucose monitoring systems, injection and infusion systems, and electrical devices for health and well-being. Health and medicine-related topics were the main focus of the publishers. The patient representatives were members of non-profit organisations, and the healthcare professionals were experts in different fields such as diabetologist and ophthalmologist. Healthcare, medical informatics, and health management were the areas where the scientists conducted research.

**Table 1:** Stakeholder characteristics

Stakeholder	Women		Men		Total	
	*n*	%	*n*	%	*n*	%
Company employees	8	16	15	30	23	46
Employees of health insurance providers	1	2	4	8	5	10
Scientists	2	4	2	4	4	8
Publisher	2	4	2	4	4	8
Healthcare professionals	2	4	2	4	4	8
Patient representatives	2	4	1	2	3	6
Other	4	8	3	6	7	14
Total	21	42	29	58	50	100

### Data compilation and content analysis

The World Café took place in July 2022. We planned the full programme to last for 3.5 hours, including breaks. We organised four discussion groups, or tables, with an average of 12–13 participants in each. SÖ, MS and ME formulated the programme and shared it with the moderators of each table. CS moderated one table, and representatives of the cooperation partner, as volunteers, moderated the other three tables. The moderators conducted the sessions, displayed the stakeholders’ contributions by putting notes on notice boards and flip charts for each table through the sessions, and summarised the sessions. Thus, the attendees had the opportunity to see previous conversations and specific ideas shared.

SÖ and MS shared detailed information about the format with all participants before dispersing to the tables. Attendees visited each table one by one, according to pre-prepared lists, without any criteria, visiting all tables. We planned three sessions. The first session consisted of three stages matched with discussions about three concepts: 1. Stage, awareness; 2. Stage, digitalization; and 3. Stage, new forms of care. Participants changed tables after each stage to make new connections. The moderators provided a summary of the topics discussed at each table at the end of each stage. All tables simultaneously discussed the difficulties and challenges of dealing more effectively with diabetes, organized around the three concepts. Each participant engaged in the discussion of all the topics.

In the second session, we used three tables because each group discussion represented a concept. Before starting the session, we gave attendees time to examine the notice boards and flip charts for each concept and to generate ideas. Then, with the assistance of the moderators, participants shared potential solutions to the challenges and difficulties faced in dealing with diabetes. Each table received a summary of the session from the moderators. Our goal for the final session was to share the topics discussed and the ideas that emerged in the previous sessions with all stakeholders. Moderators recapitulated the discussions surrounding awareness, digitalization and new forms of care by identifying the existing challenges and difficulties and their potential solutions for each concept. SÖ managed the recording of all sessions and stored these securely. She also classified and stored notes on notice boards and flip charts according to the concepts, tables, and sessions. We translated into English only the quotations we included in this Viewpoint.

We transcribed all recordings into Microsoft Word files through a private transcription service. SÖ and MS checked the transcriptions for accuracy. CS transferred the stored notes to Microsoft Word documents for analysis. SÖ used MAXQDA (VERBI, version 2022.4.1) to create and conceptualise the codes and categories by applying qualitative content analysis [12]. She read word-by-word the texts to derive codes and then formed categories and subcategories from the codes. MS and CS checked and evaluated all codes and categories to strengthen reliability and minimise inconsistencies in the selected data. This approach enabled the moderators to maintain their neutrality during the sessions. It also allowed attendees to test the emerging ideas, suggestions, and concepts for consistency. We believe that the moderators’ summarization of the session’s findings and sharing them with stakeholders strengthened the validity of this study. SÖ and CS defined operational definitions for each category and provided illustrations for each category from the data (Supplementary Material Table S1). We ensured the anonymity of the stakeholders with the standardised identifier “D”, which stands for diabetes.

### Ethical considerations

We complied with all data protection requirements and recommendations of the European Code of Conduct for Research Integrity [13] and the World Medical Association Declaration of Helsinki [14] throughout the entire process.

## Results

The findings shed light on the difficulties in combating diabetes at the institutional and structural levels and how to tackle them from the perspective of actors in the fields of business, care, and science. Discussions revolved around the concepts of awareness, digitalisation, and new forms of care. We aimed to make the categories and subcategories understandable with the quotations we used in Table S1.

### Institutional issues encountered in combating diabetes

Participants highlighted the importance of prevention in discussions. They commonly addressed diversity about the population at risk and transparency in discussing awareness and new forms of care. In discussing digitalisation, participants emphasised standardisation, data management, and the importance of encouraging digitalisation.

### Prevention

On the topic of awareness, participants associated the notion of prevention with learning, early diagnosis, media, and incentives for health insurance companies. They underlined the necessity of providing regular health and nutrition education for employees in workplaces by occupational physicians, to students and instructors in educational institutions by the relevant unit, and especially to children and employees in kindergartens. To raise awareness of healthy nutrition at an early age, a particular suggestion emerged: “*You could go to kindergartens and schools and educate them about diabetes and other diseases*” (D4). Attendees agreed that health and nutrition education should also involve parents and grandparents to support activities in educational institutions. They recommended preparation and dissemination of materials about diabetes including brochures, posters, podcasts and videos. For reaching the younger generation, they suggested organizing campaigns on social media platforms to create or increase awareness about diabetes (D8, D22). They drew attention to the identifiability of risk groups and the possibility of making early diagnoses based on regular check-ups and screening programmes (D2, D15, D27). They also voiced an expectation that health insurance companies should play a pivotal role in efforts to prevent diabetes. Companies should ensure the sustainability of existing services and offer activities or measures to boost patient motivation: “*I think if you summarise it in [a few] words, then I would say patient motivation is definitely one of those keywords*” (D3).

Participants noted that digital diabetes information portals and training courses provided by relevant experts would increase accessibility to more information for more people (D2, D27). For early diagnosis and creating new forms of care, participants advocated the creation of a diabetes map of the region to identify risk groups, like ‘obese’ or ‘pre-diabetic’, to characterise patients according to the different types of diabetes and other chronic diseases caused by diabetes, or to provide an overview of the potential conditions of immigrant groups (D15). Outside the designated centres, ‘diabetes buses’ can help to reach many people as part of a mobile prevention programme, and data collected by smart sensors can provide an overall picture of the region (D3). Putting all these measures into practice will require additional budget or resolution of financial issues (D1).

### Transparency

Key issues in the transparency discussions included access to reliable information, avoiding confusing information on relevant websites and social media platforms, and including detailed disclosure of product ingredients for the consumer. Participants also indicated that, as learned during the COVID-19 pandemic, public sharing of diabetes cases can serve as an incentive for regular testing, such as the A1C test, in a family practice to prevent diabetes (D9, D12, D41). Stakeholders also suggested that images and messages similar to those on cigarette packets could warn consumers about harmful and unhealthy products.

### Diversity

The discussion on diversity and diabetes addressed gender, age groups, ethnic identities, and types of diabetes. Given Germany’s long history of immigration, participants stressed that the language barrier is influential, especially for access to and delivery of healthcare services, and for communication with healthcare providers (D7, D43). They indicated that materials on diabetes should be multilingual, in plain language that many people will understand, or with pictorial illustrations that convey meaning without depending on language proficiency: “*I think plain language is the best option. This is already done, for example, in Sweden and Denmark*” (D18).

### Standardisation

Some attendees noted challenges related to the many sorts of products for managing diabetes that can cause confusion for patients, for example sensors to check glucose levels (D11). They suggested the creation of standardised interfaces for sensors in healthcare facilities to enable the interoperability of different sensors and to create a database for each patient. Having noted that software used in healthcare services creates complexity for healthcare providers and beneficiaries in patient care processes (D7, D32), they emphasised the importance of creating compatible systems or better software to enable digital data transfer or data fusion between healthcare facilities and health insurance companies for each individual or patient.

### Data management

How to harness digital innovation to move towards a standardised digital healthcare system to improve communication and care was a major topic. Almost all participants noted concerns about data management and data protection. They also discussed how to ensure transparent data management and privacy (D12, D15). According to some, convincing individuals that their personal data will be protected and that IT has been developed to address potential issues could minimise potential concerns. Many shared a perception that current data management regulation restricts digital innovation: “*What we have is restrictive data management, which is different from data protection. And what we need is transparent data management, so that we know what people are doing with it and what they are allowed to do with it*” (D12).

### Encouraging digitalisation

Participants indicated that digital applications should be ‘user-friendly’ for all age groups (D4). As currently only younger patients with diabetes seem motivated to use them, they suggested raising awareness about the advantages of digital applications in all age groups. The vast majority emphasized the special importance of encouraging the older generation to use digital applications: “*Yes, let’s take a look at patients again in terms of digitalisation. We currently have an old structure. Are they ready to implement this digitalisation? The doctor said very clearly: ‘How do we get people to do this?’”* (D11).

### Structural issues encountered in combating diabetes

Discussion focused on the limited infrastructure for implementing digital approaches and the lack of experts likely to be available in the future for the growing number of people with diabetes. The most prominent point raised about new forms of care related to the need for centres accessible to patients with diabetes and their relatives, or others who want information about diabetes. Digitalisation of information systems across Germany can help if digital innovations provide standardised electronic patient applications and accompany the development of the essential IT infrastructure in urban and rural areas.

### Lack of experts

Given the growing population and estimated numbers of people likely to be diagnosed with diabetes in coming years, discussions on awareness and new forms of care and the lack of experts dominated participants’ concerns at the time (D2, D17, D37) and for the future: “*We’ll have a lack of [diabetologists]. In any case, we have to consider that. It would be good to have more diabetologists now. We have to see: how can we manage with less?*” (D5). Several participants suggested overcoming the insufficiency of diabetologists through training courses for family physicians along with boosting motivation for practicing diabetologists and assistants (whose workloads are increasing), and encouraging medical students to specialise in this field.

### IT infrastructure

Efforts for digital innovation in healthcare (telemedicine, digital learning, diabetes information portals, and gamified diabetes apps) raised a question about whether the infrastructure is equipped for use of information and communication technologies (D2). Participants commonly believed this might not be possible in private households, especially in rural areas: “*There is no point in offering digital solutions if I don’t have the opportunity to use them here in the countryside*” (D25). Therefore, Germany needs to resolve infrastructure constraints simultaneously with providing incentives for digitalisation.

### Information system

Participants placed a high value on increasing the use of an electronic medical record system for recording and storing the information of individuals who benefit from healthcare services –digitally provided to authorised users (D15, D21). One suggestion emerged for the creation of a comprehensive app with general information about diabetes specialists and care options with means to manage the treatment plan of each diabetes patient.

### Digital approaches

Participants focused on using information and communication technologies to alleviate challenges facing diabetes patients. Examples included: telemedicine, diabetes telephone services, diabetes information portals, and digital self-help fora (D4, D12, D48). Attendees also stressed that digital innovations should be ones easily integrated into the everyday lives of these patients: “*The digital solution must be well thought-out. And this is where most of them fail. There are good digital ideas, but none of them is integrated into everyday life*” (D11).

### Points of contact

Based on recent experience with COVID-19 testing centres, one recommendation called for establishing similar points of contact for early diagnosis, access to reliable information, patient follow-up, and diabetes disease management. Some also suggested offering mobile services via diabetes buses and increasing the accessibility of healthcare services and the availability of healthcare resources (D9, D14). Attendees noted these centres could act as ‘buffer zones’, easing the burdens on outpatient facilities and serving patients with diabetes from a central location. How to finance these centres, however, remained to be answered.

## Discussion

The diabetes-related focus group discussions of healthcare sector actors from the state of Rhineland-Palatinate provided a view of the situation nationally and insights for sharing with new contacts among additional stakeholders. Emergent digital health interventions to minimise the effects of the consequences of the COVID-19 pandemic may have influenced participants’ focus on digitalisation as digitalisation has, or should, inform more fully treatment, care, prevention, early diagnosis, and equipment [9]. Limitations in the extent of digital literacy among healthcare providers, and, in particular, among patients and informal caregivers explain the suggestion to encourage older people to pursue digitalisation [15, 16]. Insufficient digital health literacy likely weakens healthcare systems, inhibits access to care, and adversely impacts individual health status while increasing health-related expenses that will not be financially sustainable by healthcare systems [17].

Our findings demonstrate the need for a diabetes map of Germany and highlight the importance of directing prevention strategies primarily towards risk groups. And they suggest that targeting group-oriented, culture- and gender-specific approaches may facilitate development of more sensitive prevention and intervention models and programmes [18, 19]. Hybrid learning modules designed for varied age groups, social media campaigns, and activities promoting self-management or self-motivation are the primary prevention initiatives suggested to improve understanding of diabetes. The COVID-19 pandemic demonstrated how social media platforms have been instrumental in raising public health awareness and promoting prevention [20, 21]. Social media campaigns allow public health authorities to enhance awareness by reaching target populations with brief messages on positive behavioural changes and disease prevention [21]. Self-management, including disease monitoring of planned physical activity, blood glucose levels, and adherence to medication plans can contribute to health for those with diabetes [22]. Studies show that readily available digital therapeutic mobile apps promote physical activities that strengthen self-management and improve individuals’ quality of life [9, 23].

Healthcare facilities process patient medical data daily and share it among themselves. Protecting patient privacy and security from malicious users with information security mechanisms is a top priority for healthcare facilities [24]. Other important factors, also emphasised by attendees, include the pursuit of transparent information and data management policies to build a stronger sense of trust between beneficiaries and service providers [25]. Service providers’ use of compatible systems or standard software can minimise confusion among patients.

Although our focus group participants strongly favoured digital innovations, they also believed the infrastructure was not yet sufficient to make rapid progress. Differences in urban and rural infrastructure systems reinforced this perception. If not well managed, digital approaches can lead to an unequal impact on rural versus urban populations, and on more advantaged ones versus vulnerable groups (minorities and the elderly and patients of low socioeconomic status), and deepen health inequalities across regions [26]. Our findings also indicated that testing facilities, successfully implemented during the pandemic, will be influential in tackling early diagnosis and accessibility of information and care for diabetes. The finding of demand for the widespread use of a standardised electronic patient record system across Germany aligned with previous studies [27].

### Limitations

The average number of stakeholders per table limited the likelihood of each being involved in each discussion table, given the time scheduled for the sessions. The length of the programme may have occasionally affected the concentration and focus of the attendees. The participants represented only the healthcare sector in the federal state of Rhineland-Palatinate, thus the group did not represent the healthcare sector across all of Germany.

We placed high priority on diversity in the healthcare sector when we selected participants, and our study provides an overview of the challenges at the institutional and structural levels in dealing with diabetes in Germany, and suggests specific, feasible solutions. We believe it will encourage healthcare sector representatives to build on suggestions generated and encourage more multidisciplinary co-operation. Future research should explore how digital innovations can enhance coherent health data transfer and the use of communication technologies.

## Conclusions

Managing diabetes-related challenges can be improved using insights from many fields, including education, health, information technology, science, and manufacturing, as well as through coordination among relevant ministries at the regional and national levels. Demands for digitalisation bring with them issues beyond the inadequacy of current IT infrastructure and information systems, such as digital (health) literacy, health inequality, data management and protection, and transparency. Concerns about privacy should be addressed by formulating privacy policies and assuring confidentiality to eliminate ethical risks and build public trust. Avoiding health inequalities caused by digital transformations is likely to be achieved through development and use of new, diversity-sensitive intervention strategies.

## Supplementary Information

Below is the link to the electronic supplementary material.

Supplementary file1 (DOCX 162 kb)

## Data Availability

Not applicable.
