# Supplementary material for: Innovative approaches in discussions of diabetes among healthcare sector actors in Germany
Source: J Public Health Policy. 2024 Jul 13;45(4):610–22. doi: 10.1057/s41271-024-00509-x (PMC11609090; doi:10.1057/s41271-024-00509-x)
Supplement: Supplementary file 1 — Supplementary file1 (DOCX 162 kb) [file 41271_2024_509_MOESM1_ESM.docx]

*Viewpoint*

Innovative approaches in discussions of diabetes among healthcare sector actors in Germany

Sabahat Ölcer ^1,2,3^*, Maike Scheipers ^3^, Manfred Erbsland ^3^, Constanze Sharma ^3^

^1^ Clinic for Palliative Medicine, University Medical Center Göttingen, University of Göttingen, Göttingen, Germany

^2^ Department of Human-centred Technological Development, Institute of Computer Science, Ruhr West University of Applied Sciences, Bottrop-Campus, Germany

^3^ Institute for Management, Economics and Care in the Health Sector, Ludwigshafen University of Business and Society, Ludwigshafen am Rhein, Germany

***** Correspondence:

Dr. Sabahat Ölcer

Clinic for Palliative Medicine, University Medical Center Göttingen, University of Göttingen, Göttingen, Germany

Email: [sabahat.oelcer@med.uni-goettingen.de](mailto:sabahat.oelcer@med.uni-goettingen.de)

**Supplementary Material**

**Table S1** Categories, subcategories and illustrative quotations from discussions

| Categories | Level | Subcategories | Illustrative quotations |
| --- | --- | --- | --- |
| Awareness | Institutional level | Prevention | “You could actually see something [about the COVID-19 pandemic] on all platforms. It was being propagated by the Ministry of Health, and you were being educated about how you should behave and about vaccination and things like that. Especially with young people; if you do it in a rather unconventional way on social media, for example on TikTok, Instagram or things like that, it can have an impact. And you can see that the awareness increases at a very early stage.” (D8)  “We could, for example, also take care of such a screening programme.” (D15)  “I think we are all talking about the same thing. But prediabetes, which you can actually diagnose, can be filtered.” (D27)  “Only a very small percentage of patients undertake this preventive examination. Because diabetes begins between the ages of 40 and 50. You’re not yet sick at that age; you consider yourself healthy.” (D2)  “You can do this on YouTube, maybe via YouTube, or advertising or something.” (D22) |
|  |  | Transparency | “Let’s take a look at cigarette packets. They’re very shocking; the images are actually an exaggeration. I think that if you do a public relations campaign, you should really create transparency about the long-term effects, and show people what they could be.” (D41)  *After talking about data protection*: “But we still need more information transparency.” (D12) |
|  |  | Diversity | “Germany is becoming increasingly international. Migration! One example: in Frankfurt, 80 per cent of residents have an immigrant background. Many families there don’t speak German at all. And the level of education is often low. I think that is also a difficulty in making counselling accessible to such people. It will probably be necessary to address them personally.” (D7)  “I’ve always wished to have a picture book. A picture book where everything is illustrated. Quite systematically, which I can show to everyone without using language, with little pictures. And then it doesn’t matter [where they’re from]. I can point to it in this book and show the information, so we can have non-verbal communication.” (D43) |
|  | Structural level | Lack of experts | “Now more and more people are saying that ... we no longer have any diabetologists or that we no longer have any doctors. And that the patients are on their own” (D17)  “So really, to look at it, there are many forms, care support points and many things that are already very successful, but because of the lack of skilled workers and other matters that we have, and because of the digitalisation issues, we say, but there is a task we really want to do. What is there? Where are the problems?” (D2) |
| Digitalisation | Institutional level | Prevention | “Ms. D7 has said now. I have the diabetes information portal. If I start from the beginning, it is so holistic in prevention that it provides new preventive measures. Maybe a little bit of self-detection of diabetes symptoms or risk.” (D2)  “The training courses that are already currently available and are also provided digitally for people who say it is now much more practical from home.” (D27) |
|  |  | Standardisation | “Well, [our company] has the same problem; there are too many interfaces. For example, for a hospital setting, what software do they use there? For example, sensors? If I’m manufacturing, I even have to find out what software is being used now. Can I offer [the product] there? So even as a manufacturer you have to look in other areas, and see if there are really global interfaces.” (D11)  “Yes, that’s true. Right now, someone/everyone is making some kind of software. And the hospitals don’t know what’s on the market, and the software companies don’t know what they’re doing either.” (D32)  “There are many different software solutions, and we don’t have a single digital solution.” (D7) |
|  |  | Data management | “And then comes the issue of data protection. Data protection is also paramount.” (D15) |
|  |  | Encouraging digitalisation | “What I said was that each patient has an individual decision whether to use a system or not? Or if I don’t want to use it, then what?” (D4) |
|  | Structural level | IT infrastructure | “Yes, yes! We have heard that before. Even if you have a good infrastructure, then you notice that many are left behind by such telemedicine solutions, because we simply don’t have the equipment and the telephone reception for it.” (D2) |
|  |  | Information system | “Yes, like designing an information system that will connect different specialists, hospitals and patients. Making an app where someone says you aren’t allowed to eat this or that food.” (D15)  *On electronic patient records*: “Access to their cloud [a cloud-based health tracking and monitoring system] is created by reading the patient’s card and password. You can get this information from the cloud, including from the federal drug agency.” (D21) |
|  |  | Digital approaches | “My topic is affordable telemedical care.” (D48)  “I wrote it here: hybrid use of digital methods and real user interfaces. Because I believe that such a smartphone and tablet is not suitable for everyone.” (D4) |
| New forms of care | Institutional level | Prevention | “We have a patient; we have patients with Type I and Type II diabetes. But let’s break it down a bit more. So I have patients who are healthy, but at risk of developing diabetes in the future. I have already diagnosed pre-diabetics who have a very high probability of developing diabetes. And I have people with diabetes who don’t yet have any complications and then gradually develop this serious condition. This means differentiating individual forms of diabetes and developing individualised care offers for the relevant areas.” (D15)  “What we don’t yet have is the issue of financing new forms of care. We need a new form of financing, so to speak.” (D1)  “Yes! In my opinion, for example, you can start with a much earlier diagnosis. Of course, we also present the data because the possibility of working with these sensors can also give you an overall picture [of the patient].” (D3)  “Obviously, an early diagnosis! Yes, of course! Early diagnosis! We also have a very, very high number of undiagnosed cases in Germany; they can be identified through screening and, above all, simply.” (D27)  “This means that everyone is actually saying that children need to be trained [to understand the causes of diabetes]” (D4) |
|  |  | Diversity | “Currently, children with no knowledge of German do not have a teacher unless they are hired. ... And then you think: which of the 40 children can you put in the normal class? And the children are not able to factually follow the lessons in any way. This is bad. How can we integrate and provide healthcare if the prerequisites are not provided?” (D43) |
|  |  | Transparency | “I can’t get the information I need from the shopping centre so that I can [decide whether to] buy [the product] … I look at the picture, and it looks good. I buy it. And then I look at the information written in small letters. You know, any substance up to/under 100 grams doesn’t require a declaration of how much sugar is included. How much... whatever! So these little bespoke things in these little packets, they are deliberately made that way. Because you don’t need to label them.” (D9) |
|  | Structural level | Contact points | “If we are in rural areas where digitalisation is not possible, if a unit is also established as digitalisation, then we can be mobile. Or the urban area looks more realistic, because there are connected public transports, and people can easily go from A to B.” (D9)  “Should it be something stationary, something digital, for people with an immigrant background or for the elderly? Where might you need [a service]? Something mobile; something fixed?” (D14) |
|  |  | Digital approaches | “A platform for digital information events! Something like that. Of course, it can also collect data.” (D12)  “This will be an offer for telephone digital care for therapy support.” (D4)  “I think that’s what diabetology practices are doing now. Digital telemedicine, semi-telephone support! I think that’s very good.” (D48) |
|  |  | Lack of experts | “Well, all I heard was that they [diabetologists] are very hard to get. They exist, but they’re few and far between and in high demand.” (D37) |
